# Temperature, topography, soil characteristics, and NDVI drive habitat preferences of a shade‐tolerant invasive grass

**DOI:** 10.1002/ece3.6735

**Published:** 2020-09-23

**Authors:** Anna K. M. Bowen, Martin H. H. Stevens

**Affiliations:** ^1^ Department of Biology Miami University Oxford Ohio USA

**Keywords:** biological invasions, ecological niche model, exotic plant species, Maxent, *Oplismenus undulatifolius*, species distribution model

## Abstract

**Aim:**

Despite the large literature documenting the negative effects of invasive grasses, we lack an understanding of the drivers of their habitat suitability, especially for shade‐tolerant species that do not respond positively to canopy disturbance. We aimed to understand the environmental niche and potential spatial distribution of a relatively new invasive species, wavyleaf basketgrass (*Oplismenus undulatifolius* (Ard.) Roem. & Schult, WLBG) by leveraging data available at two different spatial scales.

**Location:**

Mid‐Atlantic region of the United States.

**Methods:**

Maximum entropy modeling (Maxent) was used to predict the habitat suitability of WLBG at the regional scale and the landscape scale. Following variable evaluation, model calibration, and model evaluation, final models were created using 1,000 replicates and projected to each study area.

**Results:**

At the regional scale, our best models show that suitability for WLBG was driven by relatively high annual mean temperatures, low temperature seasonality and monthly range, low slope, and high cumulative Normalized Difference Vegetation Index (NDVI). At the landscape scale, suitability was highest near roads and streams, far from trails, at low elevations, in sandy, moist soil, and in areas with high NDVI.

**Main Conclusions:**

We found that invasion potential of this relatively new invader appears high in productive, mesic habitats at low slope and elevations. At the regional scale, our model predicted areas of suitable habitat far outside areas where WLBG has been reported, including large portions of Virginia and West Virginia, suggests serious potential for spread. However, large portions of this area carry a high extrapolation risk and should therefore be interpreted with caution. In contrast, at the landscape level, the suitability of WLBG is largely restricted to areas near current presence points, suggesting that the expansion risk of this species within Shenandoah National Park is somewhat limited.

## Graphical abstract

We aimed to understand the environmental niche and potential spatial distribution of a relatively new invasive species, wavyleaf basketgrass, by leveraging data available at two different spatial scales. We found that invasion potential of this relatively new invader appears high in productive, mesic habitats at low slope and elevations. At the regional scale, our model predicted areas of suitable habitat far outside areas where WLBG has been reported, including large portions of Virginia and West Virginia, suggests potential for spread. In contrast, at the landscape level, the suitability of WLBG is largely restricted to areas near current presence points, suggesting that the expansion risk of this species within Shenandoah National Park is somewhat limited.

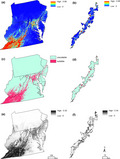

## 1 INTRODUCTION

Drivers of invasive plant species establishment have been a central topic in ecology for several decades. The importance of this topic is relevant not only for monitoring and management, but also vital to our basic understanding of invasive plants and their niches. Much of the research in this field has focused on local‐scale determinants of species or community characteristics, such as propagule pressure, species traits, resource availability, and disturbance (Burke & Grime, 1996; Davis, Grime, & Thompson, 2000; Eschtruth & Battles, 2009; Fridley et al., 2007; Luken, 2003; Meekins & McCarthy, 2001). At the landscape scale, however, there has been limited progress in determining characteristics associated with increased susceptibility of communities and ecosystems to invasions (Hayes & Barry, 2008; Williamson & Fitter, 1996).

In some cases, the size of a species' geographical range size and its native climate can predict invasiveness (Hayes & Barry, 2008; Williamson & Fitter, 1996). However, it is unclear which specific environmental tolerances at the landscape scale determine invasive establishment. By focusing on a single species or plant functional type, we can begin to refine our understanding.

Invasive grass species have caused some of the most destructive and widespread invasions globally (Barden, 1987; Brewer, 2011; D'Antonio & Vitousek, 1992; D'Antonio, Hughes, Mack, Hitchcock, & Vitousek, 1998; Pysek, 1998; Williams & Baruch, 2000; White, Campbell, & Kemp, 1997) but we lack a clear understanding of the landscape variables that contribute to their potential distribution and the likelihood that areas will serve as suitable habitat. Even less is known regarding the suitability of shade‐tolerant grass species, as most studies on invasive plants focus on species that respond positively to disturbance (Martin, Canham, & Marks, 2009). Understanding these landscape variables will not only aid in determining the factors associated with invasion, but also in predicting potential spread and sites for detection and eradication (Beauchamp et al., 2013; Brown, Spector, & Wu, 2008; Guisan & Thuiller, 2005; Jarnevich & Reynolds, 2011; Lemke, Hulme, Brown, & Tadesse, 2011; Liang, Clark, Kong, Rieske, & Fei, 2014; Yang, Kushwaha, Saran, Xu, & Roy, 2013). From a management perspective, effective early prediction and rapid response can mean the difference between effective eradication and the tedious, long‐term management of a harmful invader (Jarnevich, Holcombe, Barnett, Stohlgren, & Kartesz, 2010; Rejmánek, 2000). In addition, monitoring areas of high invasion risk can increase the probability of detection (Lodge et al., 2006).

Ecological niche models (ENMs), also known as species distribution models (SDMs) or habitat suitability models, are a widely used method to describe the potential extent of invasive species spread, highlight priority locations for future surveying, and overcome sparse presence and absence data (Beauchamp et al., 2013; Jarnevich & Reynolds, 2011; Lemke et al., 2011; Liang et al., 2014; Padalia, Srivastava, & Kushwaha, 2014). They can be used to estimate current ranges, expected habitat suitability of an expanding species, change in suitability over time, and to estimate niches (Warren & Seifert, 2011).

Despite the popularity of using ENMs for predicting invasive species’ future distributions, extrapolating presence to a new geographical range introduces many challenges. First, modeled climate niches of invasive plant species may shift when they are introduced to new continents, with many niches shown to expand in size (Atwater, Ervine, & Barney, 2018). Lack of niche conservatism might be the result of changed biotic interactions, such as a release from predators or competitors (Keane & Crawley, 2002), niche evolution (Jiménez‐Valverde & Lobert, 2011), or acclimation to new environments (Duncan, Cassey, & Blackburn, 2009; Pearman, Guisan, Broennimann, & Randin, 2008). Second, transferring ENMs to different climate scenarios or new ranges can result in problematic extrapolations and must be handled with great care (Elith et al., 2011; Ervin & Holly, 2011; Warren & Seifert, 2011). Models created within the geographic range of presence localities, therefore, are more reliable because correlations between environmental variables tend to be consistent in that range (Elith & Leathwick, 2009). In addition, the use of background samples for pseudo‐absences in the invaded range is problematic because the species may still be expanding in extent (Rodda, Jarnevich, & Reed, 2011). Many studies have attempted to address some of these challenges by restricting the area from which background points can be selected and from the use of expert opinion (Elith, Kearney, & Phillips, 2010; Mainali et al., 2015; Murray et al., 2009; Padalia et al., 2014). Despite these limitations, ENMs remain one of the only tools available to overcome sparse data, understand drivers of invasion, and make predictions regarding potential invasive ranges from a landscape perspective (Elith et al., 2006; Jarnevich & Reynolds, 2011; Lemke et al., 2011).

To date, there are very few ENMs for shade‐tolerant grass species, and these studies reveal that a wide variety of environmental variables might drive the distributions of these species. These varied results may stem from variation in species' niches or the variation in techniques and environmental variables being used. Both Bush (2015) and Lopez‐Alvarez et al. (2015) revealed that annual precipitation was an influential variable in their models. Lopez‐Alvarez et al. also reported that temperature seasonality and mean diurnal range were highly contributing variables, while Bush found that maximum yearly temperature was the highest contributing variable and Beauchamp et al. (2013) found that annual mean temperature was a highly contributing variable. Elevation contributed highly to ENMs by Beauchamp et al. (2013) and Bush (2015), while distance to hydrologic features also contributed highly to Bush's (2015) ENM. In Beauchamp et al.'s model, soil classification was the highest contributing variable, while Ervin and Holly (2011) found that canopy cover, silt content, and cation exchange capacity were the highest contributing variables in their model of the invasive cogongrass.

**FIGURE 1:**
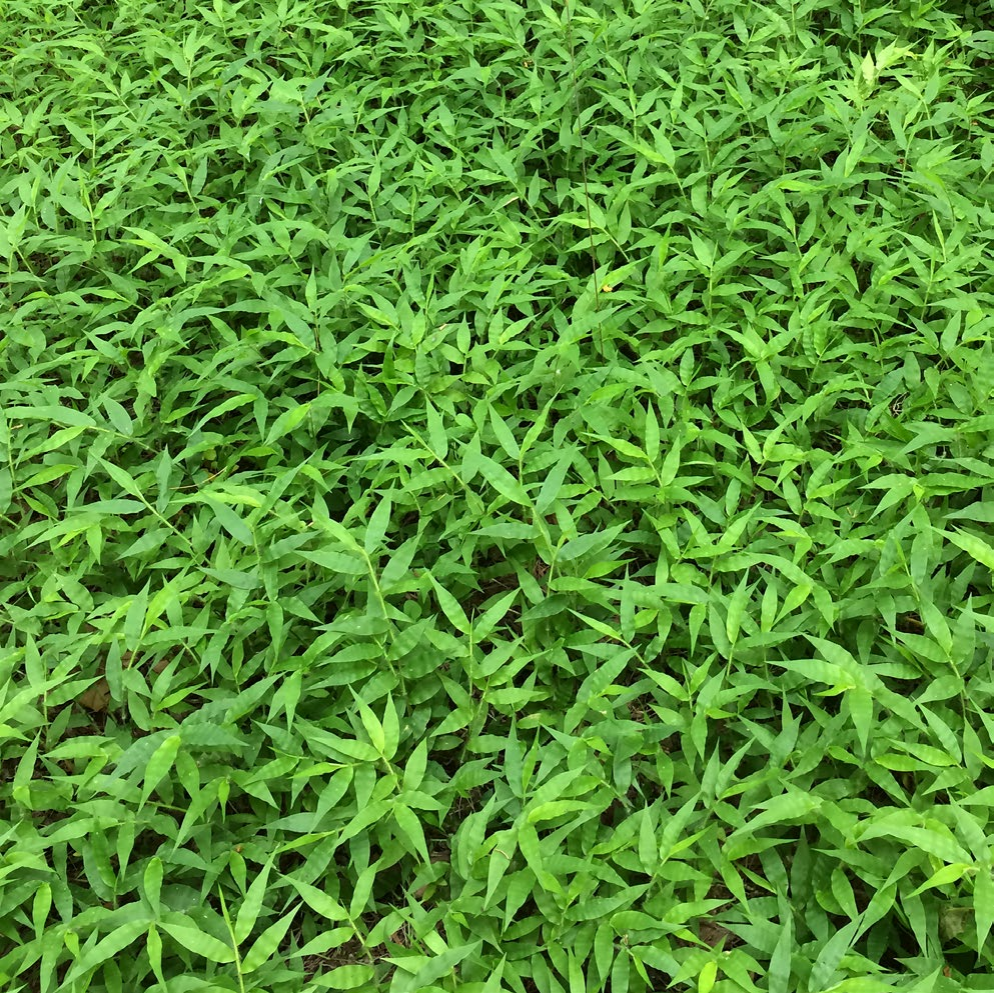
Image of *Oplismenus undulatifolius* (wavyleaf basketgrass, WLBG). Photograph by Anna Bowen

Wavyleaf basketgrass (*Oplismenus undulatifolius* (Ard.) Roem. & Schult) (WLBG) is a relatively new invasive grass species native to Europe and Asia that was introduced to the United States in 1996 near Baltimore, Maryland (Beauchamp et al., 2013; Peterson et al., 1999) (Figure 1). There is a growing concern for the potential negative effects this species may have on eastern forests. Many have observed its invasion into shaded, undisturbed understories and its potential for both long‐distance dispersal due to its sticky spikelets and extensive short‐distance dispersal via stolons (Beauchamp et al., 2013; Scholz, 1981). The USDA's weed risk assessment has classified this species as high risk in terms of establishment, spread, and impact potential, but this includes a high level of uncertainty (USDA, 2012).

Beauchamp et al. (2013) evaluated WLBG's habitat suitability with Maxent and found that suitable habitat was concentrated around known invasion sites. They also found that 1% of their study area (77,000 ha) was predicted as highly suitable. Their best model for predicting WLBG suitable habitat contained all 24 of their environmental variables, but annual mean temperature, elevation, and soil classification were the top contributing variables.

Despite the valuable insights gained from Beauchamp et al.'s model, new insights into the suitable habitat of WLBG can be gained with the addition of new data and modeling techniques. The number of WLBG presence points has more than quadrupled since 2013 (2,364 vs. 505) (EDDMapS, 2019), which suggests that the variety of environments that have been invaded has increased, leading to more insightful predictions about the factors influencing WLBG invasion. In addition, techniques such as multiple model replicates (Jarnevich & Reynolds, 2011; Liang et al., 2014; Phillips, Anderson, & Schapire, 2006), restricting the area from which background points can be selected (Elith et al., 2010; Jarnevich & Reynolds, 2011; Mainali et al., 2015; Vanderwal, Shoo, Graham, & Williams, 2009), reducing model dimensionality by dropping correlated variables (Cobos, Peterson, Barve, & Osorio‐olvera, 2019; Elith et al., 2010; Lemke et al., 2011; López‐Alvarez et al., 2015), the use of AIC for model evaluation (Warren & Seifert, 2011), and investigating combinations of regularization parameters, feature classes, and sets of predictors (Cobos et al., 2019; Peterson, Cobos, & Jim, 2018) can greatly aid in model fitting and evaluation.

We were also interested in investigating WLBG habitat suitability on more than one spatial scale. The dominant methodology for ENMs relies largely on climatic variables (Elith et al., 2010; López‐Alvarez et al., 2015; Phillips et al., 2006), but at finer spatial scales these climatic predictors may not be informative or cannot be used due to their coarse spatial resolution. Predictors such as soil pH, soil texture, and elevation may, therefore, reveal patterns regarding suitability that cannot be gleaned from climatic variables alone. In addition, land managers wishing to better understand whether they can expect a species invasion would benefit from research regarding what factors are important at finer spatial scales. Therefore, we investigated potential environmental predictors at both a regional and landscape scale with the goal of better understanding the contribution of a variety of predictors across scales.

Given how little is known about invasions by shade‐tolerant grasses, our primary objective of this study was to start building a consensus on the habitat suitability of shade‐tolerant grass species using WLBG as a study species. While suitability might increase closer to rights of way for many invaders due to increased levels of disturbance (Mortensen, Rauschert, Nord, & Jones, 2009), in contrast, shade‐tolerant grasses may rely more heavily on forest cover or soil characteristics. Our second objective was to compare and contrast drivers that are important at different spatial scales. Last, we also compare our results to those of Beauchamp et al. (2013) in order to help determine whether an increased number of presence points alter the habitat suitability of WLBG. Our overall goals for this study are to update the geographic distribution and help predict areas of future invasion with a comprehensive data set and analysis.

## 2 METHODS

### 2.1 Regional‐scale model

**FIGURE 2:**
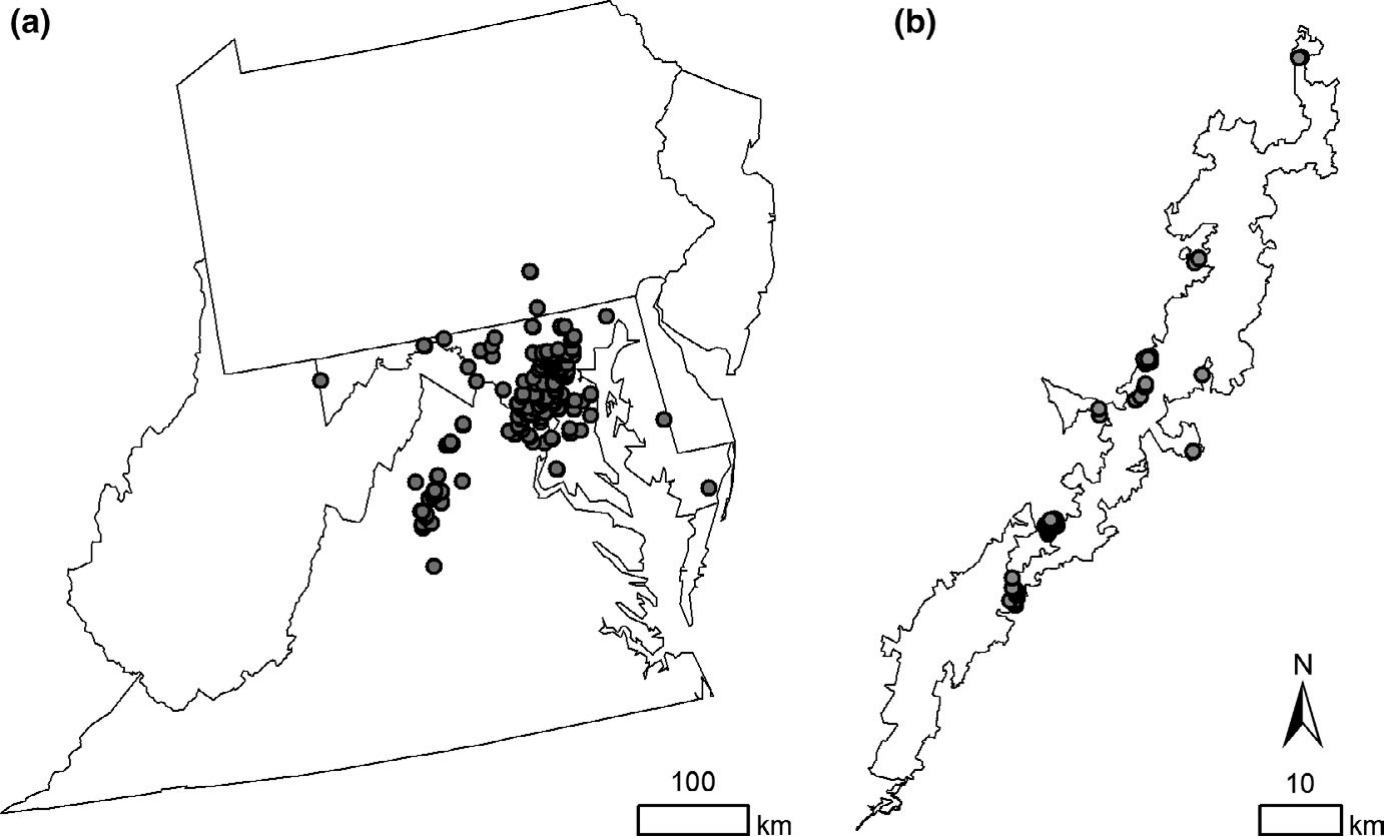
WLBG presence points (404 points) used for maximum entropy modeling at the regional scale (a) and at the landscape scale (82 points) in SHEN (b)

To create a maximum entropy model (Maxent) for WLBG at the regional scale, we first selected a study area that included Delaware, District of Columbia, Maryland, New Jersey, Pennsylvania, Virginia, and West Virginia (Figure 2a). These states were chosen because WLBG either occurs in that state or a directly adjacent state (EDDMapS, 2019).

We compiled 2,364 WLBG presence points from three sources: a 2016 survey for WLBG in Shenandoah national park (Bowen and Stevens, unpub. data), previously discovered presence points from Shenandoah park staff (J. Hughes, pers. communication), and from the early detection and distribution mapping system (EDDMapS, 2019). To reduce the amount of overfitting around spatially autocorrelated points, we spatially filtered these points down to one point per 250 m^2^ cell (Beauchamp et al., 2013; Jarnevich & Reynolds, 2011; Mainali et al., 2015), which resulted in 404 points total (Figure 2a). The minimum, maximum, and mean distance between all points was 250 m, 368,037 m, and 1,800 m, respectively.

Thirty‐eight environmental layers were obtained for predictors in Maxent and were clipped to the study area at 250 m^2^ resolution (Table S1). Predictors included climate variables from BIOCLIM v. 2 (Hijmans, Cameron, Parra, Jones, & Jarvis, 2005), phenological variables (U.S. Geological Survey, 2016), topographical variables (U.S. Geological Survey, 2013), distance to roads (USDA NRCS, 2016), distance to streams (USDA NRCS, 2016), and soil variables (Soil Survey Staff, 2018) (Table S1). Pairwise Pearson's correlations for predictor values across the entire study area were used to determine collinearity between all variables (*r* ≥ .8 or *r* ≤ −.8) (Jarnevich & Reynolds, 2011; Lemke et al., 2011). Half of these 38 variables were dropped due to collinearity, resulting in 19 variables. We chose to avoid categorical variables such as land cover and soil type in favor of continuous variables due to the tendency for Maxent to overemphasize the importance of categorical variables (M. Cobos, pers. comm.).

These 19 variables were further investigated and reduced to 10 variables to avoid potential overfitting and computational limitations (Cobos et al., 2019; Townsend Peterson, Papeş, & Eaton, 2007). This investigation was done by evaluating the output of a preliminary set of 70 candidate models and selecting the top predictors for use in final model calibration. This set of 70 candidate Maxent models contained all possible combinations of all 19 predictors, 14 regularization parameter values (0.1–1 at intervals of 0.1 and 2–5 at intervals of 1), and five feature classes (l, lq, lqp, lqpt, and lqpth, where l = linear, q = quadratic, p = product, t = threshold, and h = hinge), using the kuenm package in R (Cobos et al., 2019). The regularization parameter controls the intensity of the chosen feature class and can smooth these functions in order to prevent overfitting (Morales, Fernández, & Baca‐González, 2017). Feature classes, on the other hand, allow mathematical transformations of the data in order for complex (or simple) relationships to be modeled (Morales et al., 2017). There have been several recent calls for investigating these parameter options, as default values may not always be appropriate and do not penalize model complexity (Cobos et al., 2019; Phillips & Dudík, 2007; Warren & Seifert, 2011).

Receiver operating characteristic (ROC) curves, which measure model performance, can become inflated with over‐parameterization (Elith et al., 2010; Vanderwal et al., 2009), so the following steps were taken both in preliminary and final model calibration to avoid ROC inflation. We restricted the area from which background points were selected using a buffer indicating how far WLBG could have reached if conditions were suitable (Elith et al., 2010) using the distance between the earliest record of WLBG (K. Kyde, pers. communication) and the maximum distance from that record to another presence point. Background points (10,000) were then created within that buffer and used for model creation. 25% of presence points were set aside for model testing (Liang et al., 2014; Phillips et al., 2006). Model iterations, therefore, contained 303 training and 101 testing points.

We evaluated the model performance of these 70 candidate models based on significance (partial ROC with 500 iterations and 50% of data for bootstrapping), omission rates (*E* = 5%), and model complexity (AICc) (Cobos et al., 2019). We then used the jackknife output of the best model to select the top ten contributing variables, using the sum of the gain with only that variable and the largest gain lost without that variable. The final set of ten variables were as follows: annual mean temperature, temperature seasonality, temperature annual range, diurnal range, annual precipitation, precipitation seasonality, end of season time (EOST), time‐integrated NDVI (TIN), elevation, and slope. Temperature seasonality represents the variation in temperature across the year (standard deviation * 100), while diurnal range is a measure of monthly temperature range (mean of monthly (max temp − min temp)). EOST is the ending time of the growing season (in day of the year), while TIN is the cumulative value of NDVI from the start to the end of the growing season (unitless—based on accumulated NDVI units).

Using these 10 predictor variables, 70,910 candidate models were created, using all possible combinations of at least two of those ten variables (1,013 sets of variables), the 14 regularization parameters and 5 response feature classes as above, as well as the same background point selection and proportion of testing and training points.

Once the best model was selected using the same measures of performance as with preliminary model calibration (above), a final model was created by transferring this best model to the full study area. We ran 1,000 replicates of this model by bootstrapping 50% of the testing data. We compared the response curves for each of the following model outputs: free extrapolation, extrapolation with clamping, and no extrapolation (Cobos et al., 2019) and selected the model with the most realistic output curves, which was extrapolation with clamping. Finally, these extrapolation risks were evaluated with the mobility‐oriented parity (MOP) metric, which calculates multivariate distances between points in the projection and calibration regions (Owens et al., 2013). It is vital to understand extrapolation risks when transferring a model to a new geographical area or to a new time period to avoid making overly strong conclusions regarding predicted suitable areas (Owens et al., 2013). Extrapolation may occur in a variety of ways. Strict extrapolation occurs when values within the study area are outside the range of those within the background training area, while multivariate or combinational extrapolation occurs when values may be within the same range but represent new combinations of predictors (Owens et al., 2013).

Four maps were created with the results of final model creation: a map of mean predicted suitable habitat between the 1,000 replicates, a binary map showing predicted suitable and unsuitable habitat using the maximum training sensitivity plus specificity threshold calculated by Maxent (mean threshold value across the 1,000 runs) (Lemke et al., 2011; Liang et al., 2014), a map of standard deviation between predictions from these replicates (Jarnevich & Reynolds, 2011), and a map of extrapolation risk. In addition, the area of suitable habitat (prediction ≥ 0.224 via the maximum training sensitivity plus specificity threshold) and highly suitable habitat (prediction ≥ 0.5) (Beauchamp et al., 2013) was calculated using the mean model and the latter was compared to the area found in Beauchamp et al.'s (2013) model within an estimate of their study area.

### 2.2 Landscape‐scale model

Shenandoah National Park (SHEN) was used as a case study for a landscape‐scale model of habitat suitability for WLBG. Not only does SHEN occupy a large elevational range and a variety of forest habitats, the staff at this park have had an active interest in the spread of WLBG within the park since its discovery in 2005 (J. Hughes, pers. comm.). Over 1,000 presence points have been recorded in SHEN, both from haphazard staff surveys and from a stratified survey throughout the park in 2016 (Bowen and Stevens, unpub. data, J. Hughes, pers. comm.).

As with the regional‐scale model, presence points (1,579) were compiled and spatially filtered to one point per 30 m^2^ cell to avoid overfitting (475 points). However, initial runs of this model indicated that overfitting was likely to have occurred due to (a) the high concentration of higher prediction values immediately surrounding presence points and (b) the appearance of high spatial autocorrelation for presence points despite the filtering process. Therefore, points were further filtered to one point per 250 m^2^ cell as with the regional model, this time using the exact location of each point rather than the centroid of the 250 m^2^ cell. This filtering reduced the number of points to 82 and reduced clustering (Figure 2b). The minimum, maximum, and mean distance between all points was 38 m, 78,021 m, and 274 m, respectively.

Twelve environmental layers were obtained and clipped to the study area at a 30 m^2^ resolution (Table S1), where they were then investigated for collinearity and contribution using the methods described above. Several of these layers were also used in the regional‐scale model, but climate and phenology layers were not used due to their low spatial resolution. Distance to trails (SHEN park staff) and NDVI (Richardson et al., 2017) were added as new layers for this model. Pairwise Pearson's correlations for these 12 predictors were calculated as with the regional‐scale model, and one variable was removed (soil silt content) as it was correlated with soil sand content (*r* > .70). Using a preliminary set of 70 candidate models as with the regional model and these 11 environmental variables, the jackknife of the best model was used to identify the top ten contributing variables. One variable (soil clay content) contributed very little to the model and was removed. Background point selection was not restricted as it was with the regional‐scale model as our interest was not with extrapolation to the region but with WLBG suitability in SHEN. The final set of ten variables were distance to roads, distance to trails, distance to streams, elevation, slope, aspect, soil ph, soil sand content, soil available water storage, and NDVI.

Candidate models were then created as with the regional‐scale model by using these ten predictor variables and the same combinations of regularization parameters and feature classes (70,910 candidate models). 25% of points were restricted for testing (20 points), leaving 62 points for model training. Model evaluation was performed as above, using partial ROC, omission rates, and AICc.

A final model with 1,000 replicates was created as with the regional‐scale model, from which mean suitability, suitable versus unsuitable (prediction ≥ 0.188 via the maximum training sensitivity plus specificity threshold and 0.50, respectively), and standard deviation maps were created. The area of suitable and highly suitable habitat was also calculated for SHEN.

All final maps were created in ArcMap version 10.4.1 (ESRI, 2016). All analyses including Maxent were done in R version 3.5.2 (R Core Team, 2018) using packages factoextra (Kassambara & Mundt, 2017), kuenm (Cobos et al., 2019), rattle (Williams, 2011), raster (Hijmans, 2017), and sp (Bivand, Pebesma, & Gomez‐Rubio, 2013; Pebesma & Bivand, 2005). Candidate model creation and evaluation for the regional scale were run using the high‐performance cluster at Miami University, and model creation and evaluation took 12 and 41 days, respectively.

## 3 RESULTS

### 3.1 Regional‐scale model

**FIGURE 3:**
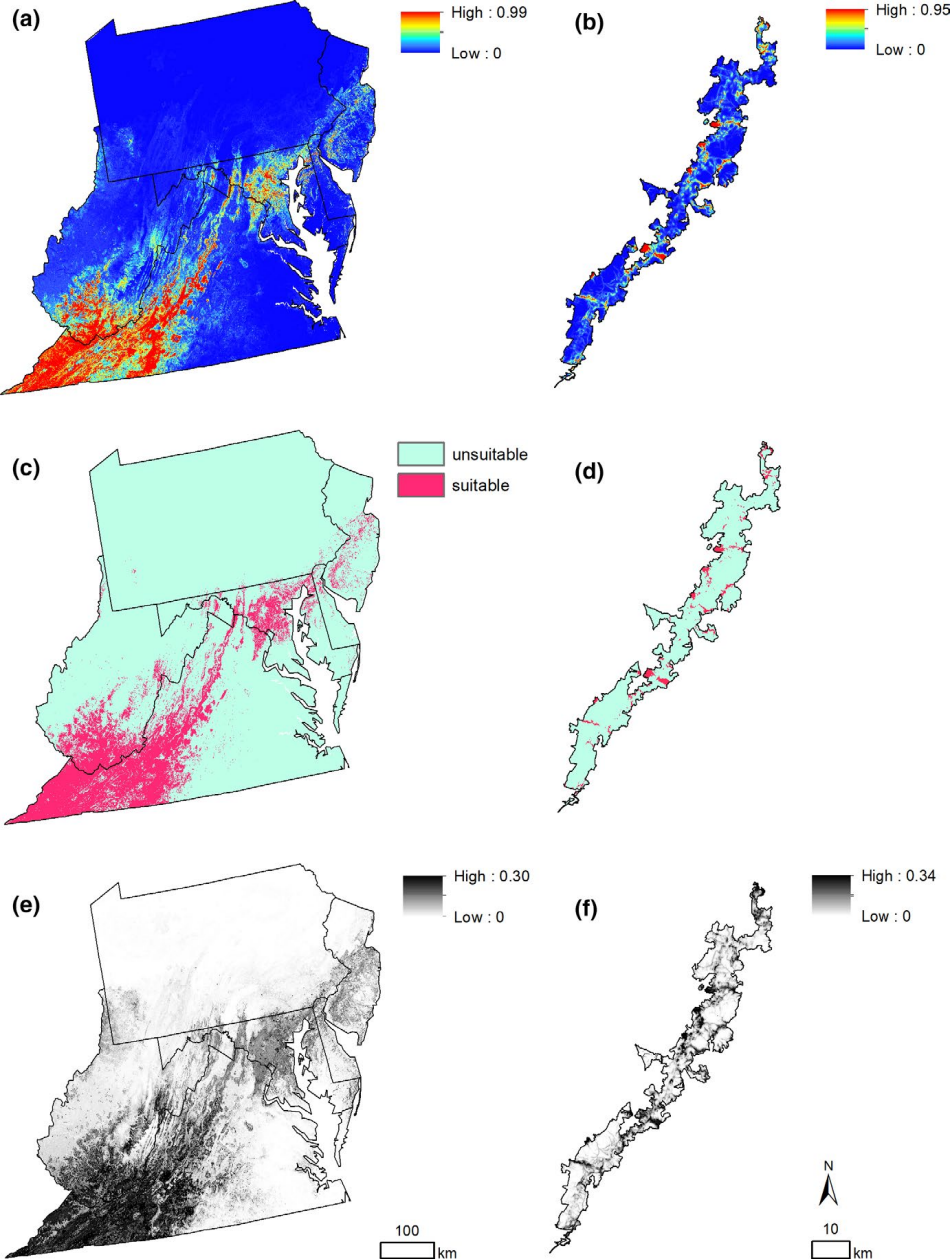
Maxent map results across 1,000 iterations for the regional‐scale model (a, c, e) and the landscape‐scale model in SHEN (b, d, f), including mean Maxent maps where warmer colors show more suitable predicted habitat (a, b), binary maps of suitable habitat and unsuitable habitat (c, d), and standard deviation maps across 1,000 model replicates (e, f)

**FIGURE 4:**
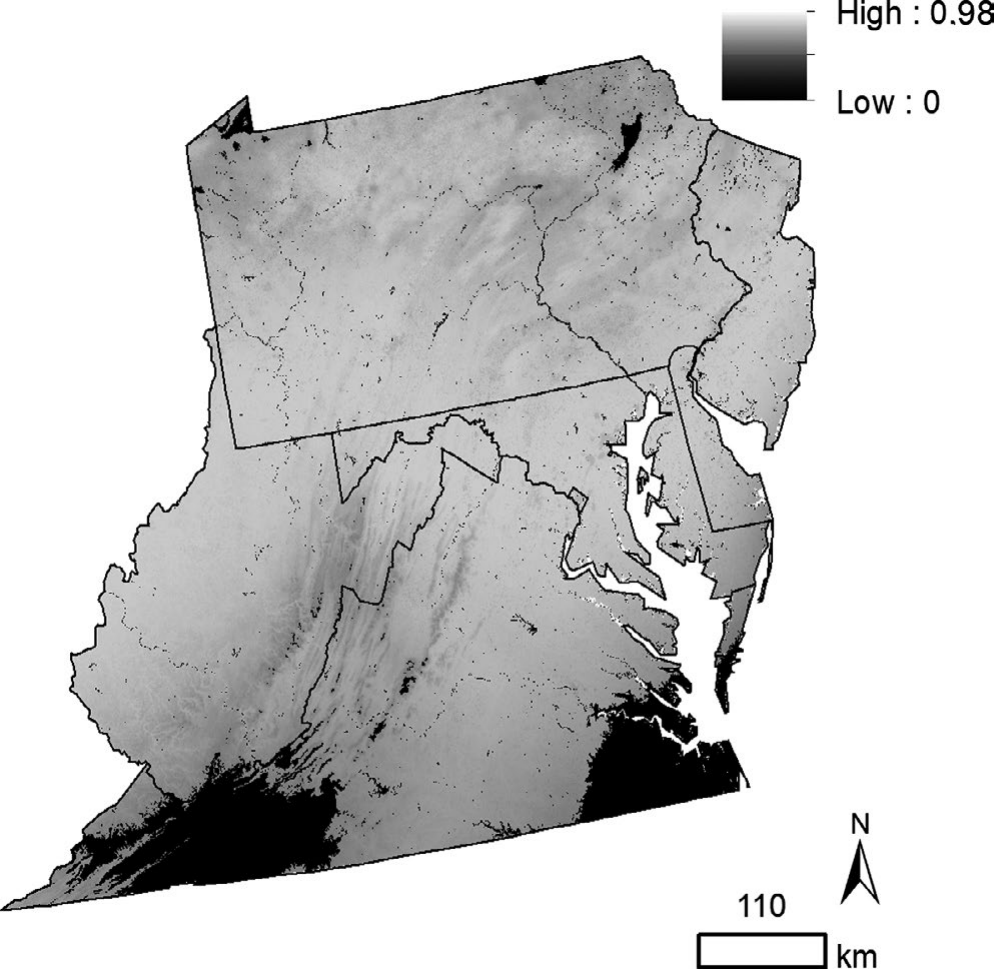
Mobility‐oriented parity (MOP) analysis results for extrapolation risk. Black areas (value of zero) indicate areas of strict extrapolation, while all other areas indicate the similarity between the projection and calibration regions. Lighter areas indicate a higher similarity between regions

Predicted suitable habitat for WLBG in the mid‐Atlantic region expanded far beyond presence points, with high predicted suitability in southwest Virginia and southern West Virginia (Figure 3a,c). A portion of the highly suitable areas in southwestern Virginia also fell within the area of high extrapolation risk according to our mobility‐oriented parity (MOP) analysis results (Figure 4) and had high standard deviations (Figure 3e), indicating lower confidence in those predicted areas. However, the majority of the study area did not have a substantial extrapolation risk. The calculated area of suitable habitat was 14.92% of the study area, while the area of highly suitable habitat was 7.25% of the study area. Within Beauchamp et al.'s (2013) study area, highly suitable habitat in our model occupied 258,035.81 ha (3.23%).

Of 70,910 candidate models with 10 or fewer predictors, the majority were both statistically significant and met the omission rate criteria (Table 1). The best model had five of the original 10 predictors: annual mean temperature, temperature seasonality, slope, diurnal range, and time‐integrated NDVI (TIN). The following variables were therefore not included in the best model: annual precipitation, elevation, end of season time (EOST), precipitation seasonality, and temperature annual range.

**TABLE 1:** Calibration results and statistics for regional‐ and landscape‐scale candidate models and best models used for final model creation

	Regional scale	Landscape scale
Candidate models	70,910	70,910
Statistically significant models	68,854	70,113
Models meeting omission criteria	43,445	55,828
Models meeting AICc criteria	1	1
Statistically significant models meeting omission rate criteria	43,201	55,825
Predictors in best model	5	7
Regularization parameter in best model	0.6	0.9
Feature classes in best model	lqpth	lqpt
Parameters in best model	95	28
Mean AUC ratio in best model	1.644	1.638
Omission rate of best model	0.05	0.05
AICc value of best model	9,917.93	1,886.55

**FIGURE 5:**
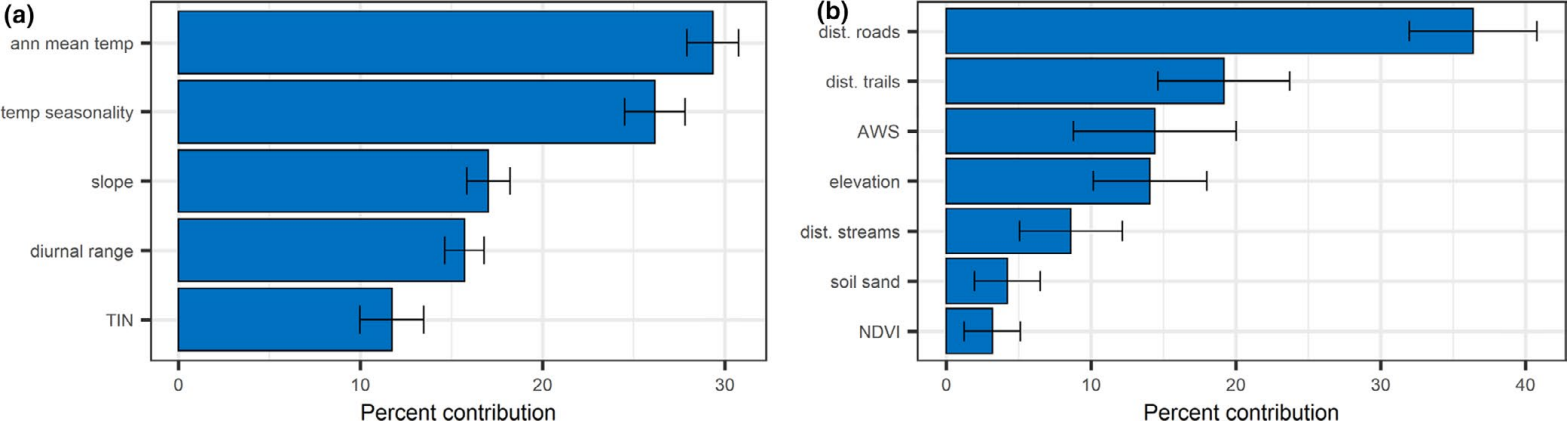
Percent contribution of variables used for final models, listed in order of mean percent contribution (± standard deviation) calculated across 1,000 model replicates for regional‐scale model (a) and landscape‐scale model (b). TIN = time‐integrated NDVI (Normalized Difference Vegetation Index) aka cumulative NDVI over the growing season, AWS = available water storage to 25 cm

The top contributing environmental variables to the final model were annual mean temperature (30.05% ± 1.45 *SD* across replicates) and temperature seasonality (24.84% ± 1.60), while the variables with the lowest contribution were slope (17.03% ± 1.21), diurnal range (16.10% ± 1.06), and time‐integrated NDVI (TIN) (11.98% ± 1.76) (Figure 5a).

**FIGURE 6:**
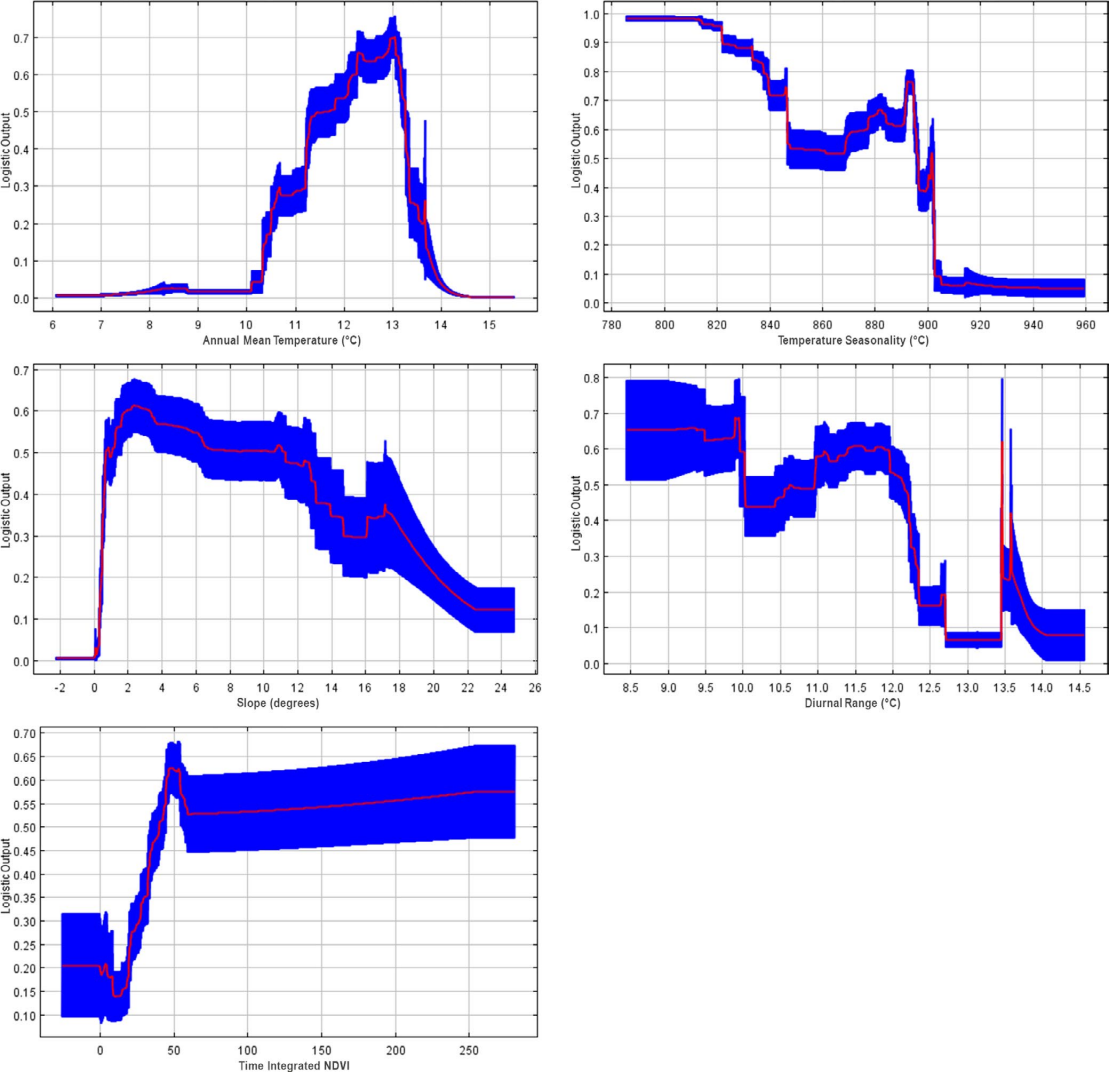
Response curves between Maxent suitability predictions (*y*‐axes) and predictors (*x*‐axes) in final models for regional‐scale model in order of decreasing variable importance. Red lines indicate means across the 1,000 replicates, with blue areas indicating ±1 standard deviation from that mean

The best model showed complex relationships between predicted habitat suitability (logistic output) and the top five predictors (Figure 6). For climate variables, suitability was highest with annual mean temperatures near 12–13°C, low temperature seasonality (800–820), while the relationship with diurnal range showed high suitability between 8.5 and 10.0 with an additional peak at 13.5. Slope showed that suitability was highest near 2 degrees with decreasing values at increasing slopes, while time‐integrated NDVI (TIN) had high suitability above values of 50, indicating areas with a high level of cumulative NDVI.

### 3.2 Landscape‐scale model

Predicted suitable habitat for WLBG in Shenandoah National Park was largely concentrated near existing presence points but extended beyond presence points in a few regions in the park, particularly in the central region (Figure 3b,d). Many areas with predicted suitable habitat, however, also had high standard deviations across models (Figure 3f). The calculated area of suitable habitat was 6.75% of the study area, while the area of highly suitable habitat was 1.05% of the study area.

As with the regional model, the majority of candidate models were both statistically significant and met the omission rate criteria (Table 1). The best model had seven of the original ten predictors: distance to roads, distance to trails, available water storage (aws), elevation, distance to streams, soil sand content, and NDVI. The variables slope, aspect, and soil pH were therefore not included in the best model.

The top contributing environmental variables to the final model were distance to roads (34.45% ± 4.26 *SD*), distance to trails (19.12% ± 4.29), soil available water storage (15.83% ± 6.00), and elevation 14.12% ± 3.55) (Figure 5). The variables with the lowest contribution were distance to streams (8.85% ± 3.38), soil sand content (4.13% ± 2.07), and NDVI (3.52% ± 1.98) (Figure 5b).

**FIGURE 7:**
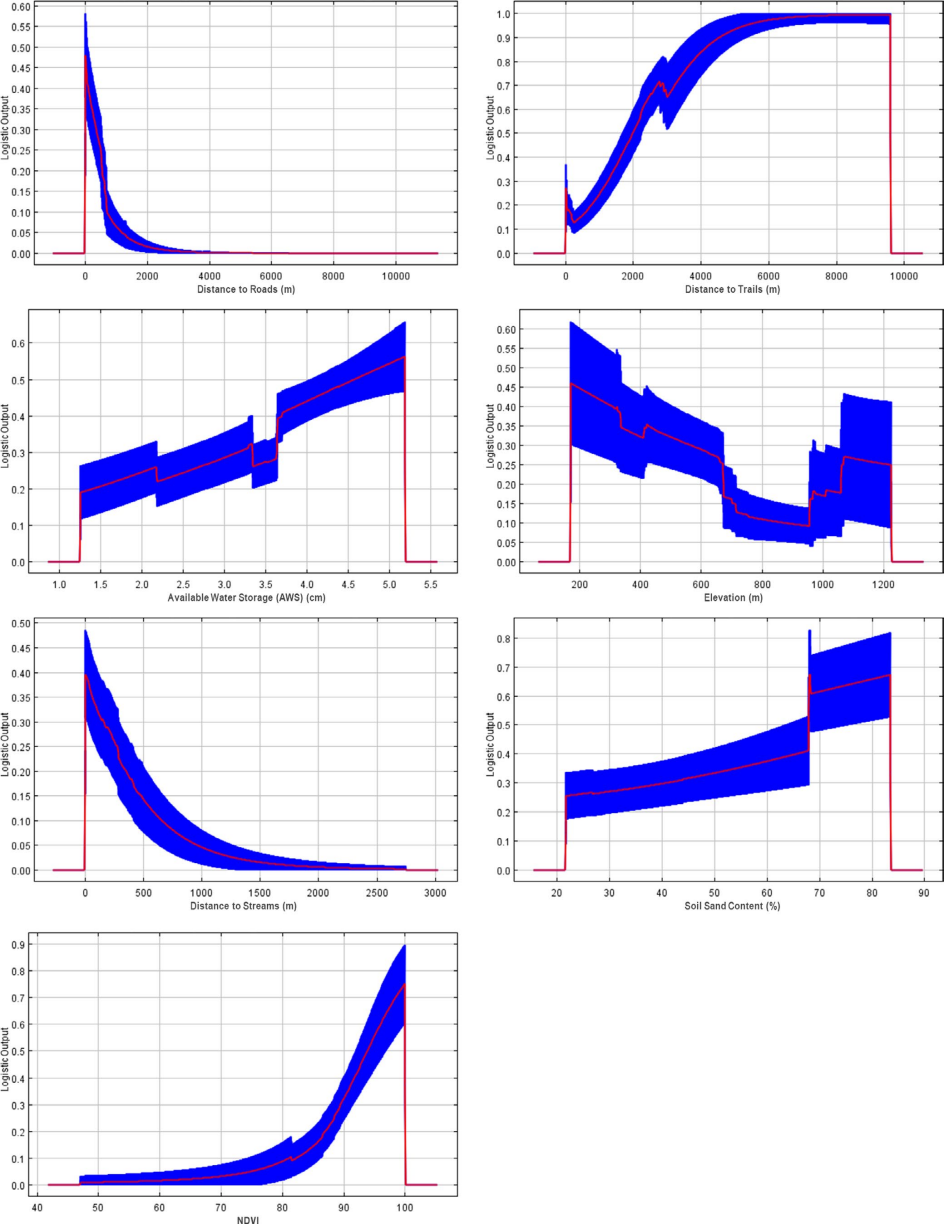
Response curves between Maxent suitability predictions (*y*‐axes) and predictors (*x*‐axes) in final models for landscape‐scale model in order of decreasing variable importance. Red lines indicate means across the 1,000 replicates, with blue areas indicating ±1 standard deviation from that mean. NDVI was scaled by 0–100 rather than 0–1 for computational ease

Habitat suitability in SHEN showed mostly monotonic relationships with our seven predictor variables (Figure 7). Suitability tended to increase strongly closer to streams and roads but had the opposite trend with distance from trails, where suitability was highest at distances in excess of 4,000 m from trails. Suitability was also higher in areas with sandy soil, with soils of at least 70% sand having the highest suitability. Suitability also increased with available water storage (aws) and NDVI, with the highest suitability in areas with at least 4.0 cm of water storage and at least 0.90 NDVI. Suitability tended to be higher at low elevations (200–400 m) and showed moderate suitability at higher elevations (1,500–2,000 m), but this included a higher degree of variation between replicates.

## 4 DISCUSSION

Our results are fairly consistent with other ENMs of shade‐tolerant grasses, suggesting a growing consensus regarding the most important drivers for these types of invaders. First, we found that annual mean temperature, diurnal range, and temperature seasonality were also important predictors in studies by Bush (2015), Beauchamp et al. (2013), and Lopez‐Alvarez et al. (2015). In addition, Beauchamp et al.'s range of 10.8–14.2°C for highly suitable area was similar to our result, where highest predicted presence was between 12 and 13°C. Second, while annual precipitation (Bush, 2015; Lopez‐Alvarez et al., 2015) was not present in the best model in this study, it was included in two of our top models at the regional scale. Third, we found elevation was included in our top landscape‐scale model as in Beauchamp et al. (2013) and Bush (2015), with low elevation being indicative of higher predicted presence in all cases. Fourth, we found some similarities between other studies with regard to soil variables. Specifically, we also found a positive relationship between presence and soil water availability (Ervin & Holly, 2011). However, we also note that Bush (2015) found a negative relationship with this variable beyond 3.5 cm of water storage. In addition, while it was not in our top model, pH was an important variable for many of our top models, as in Bush (2015). With regard to sand content, we found a decidedly positive relationship with sand content, while Ervin and Holly (2011) found inconsistent relationships between their different models. Fifth, our strong, negative relationship between predicted presence and distance to streams was also very similar to Bush (2015) for Japanese stilt grass. Finally, we found NDVI to be an important predictor at both scales, which supports the observation that WLBG is found in shaded forests (Beauchamp et al., 2013). Both Beauchamp et al. (2013) and Ervin and Holly (2011) found that NDVI or canopy cover were important predictors in their invasive grass ENMs. However, while we found that predicted presence was highest at NDVI values exceeding 0.5, Ervin and Holly showed a strong decline with NDVI exceeding 0.8. It is reassuring that even though most of these studies either included very few environmental variables or did not investigate the relationships between predicted presence with each variable, our more comprehensive approach is broadly consistent with previous studies and further refines predictions for future work.

Interestingly, while our regional‐scale model predicted a much‐expanded spread of WLBG into the region, our landscape‐scale model did not predict a wide expansion of WLBG within Shenandoah National Park. We found that much of southwestern Virginia and southern West Virginia may be highly suitable for WLBG, in addition to some areas in New Jersey at the regional scale. However, these areas also had higher standard deviation values across model replicates, and parts of southwestern Virginia have extrapolation risks and thus must be interpreted with caution. It is important to note that our result is likely an underestimation of WLBG’s suitable habitat, as it is unlikely that this species is at equilibrium with its new environment as evidenced by its ongoing expansion into the region. At the landscape level in Shenandoah National Park, suitable habitat was largely concentrated near presence points. Standard deviation among replicates also appeared to be highest near areas of predicted suitability, softening the conclusion that WLBG will spread to neighboring states. In addition, the area of suitable and highly suitable habitat in the park was relatively small (6.75% and 1.05%, respectively). This constrained range may correspond to limited potentially suitable sites within the park or the possibility that we did not successfully prevent overfitting via spatial autocorrelation. Overfitting may also have occurred in our regional model as well, but the clear lack of concentrated predicted suitability near presence points does not reveal whether this was the case or not. However, it should be noted that while WLBG has been observed in the park since 2005 (Jake Hughes, pers. comm.), this species may be far from equilibrium in this area as it does not occupy many sites across the park, suggesting that this model is likely underestimating suitable habitat.

The predictors selected for final model creation differed between the regional and landscape scales. Five of the original 38 predictors were selected in our final regional model: annual mean temperature, temperature seasonality, slope, diurnal range, and time‐integrated NDVI (TIN). Model evaluation selected a final landscape model with seven of the top ten predictors: distance to roads, distance to trails, available water storage (aws), elevation, distance to streams, soil sand content, and NDVI. While climate and phenology predictors were not used for model calibration at the landscape scale, it is curious that slope was determined to be important at the regional but not the landscape scale, while elevation held the opposite trend. To determine the strength of the finding that these variables alone were the most important for predicting WLBG habitat suitability, we also investigated the sets of predictors for the second to tenth best models for each spatial scale (Tables S2 and S3). The top four models had the same set of predictors as the top model at the regional scale, suggesting that the importance of these five predictors is robust. All top 10 regional models included time‐integrated NDVI and slope, and all five predictors were included in at least one of the top models (results not shown). At the landscape scale, we found that all seven selected predictors except for NDVI were included as predictors in all top ten models, once again indicating the strength of these predictors.

The importance of our final predictors and their relationships with predicted presence indicate the specific habitat characteristics that would be most suitable for WLBG. Regional scale results indicate that areas with high suitability for WLBG have relatively high temperatures, low temperature seasonality and monthly range (diurnal range), low slope, and high NDVI over the growing season. At the landscape scale, suitability is predicted to be highest near roads and streams, far from trails, at low elevations, in sandy, moist soil, and in areas with high NDVI. Beauchamp et al. (2013) reported that WLBG habitat suitability was high in areas with annual mean temperatures between 10.8 and 14.2°C and elevation between 15 and 193 m. We found that high WLBG suitability was associated with a very similar annual mean temperature range, while elevation seemed to have a more subdued effect, yet suitability was highest at the lowest elevations. There is a large literature citing the importance of rights of way (roads and trails) to invasive plant distributions (Christen & Matlack, 2009; Mortensen et al., 2009; Pauchard & Alaback, 2004). The results from our landscape‐scale model show that WLBG suitability was high near roads but far from trails. These two predictors, despite their high contribution to the model, should be interpreted with caution. All presence points in SHEN were relatively close to roads due to the nature of data collection within the park. In addition, the distance from one right of way feature does not indicate the distance from the other. In other words, a presence point may be far from a trail but close to a road. While distance to roads as a predictor supports the hypothesis that plant invaders establish in disturbed areas, distance to trails had the opposite prediction and supports the observation that WLBG establishes within the forest interior and thus far from human‐disturbed right of ways.

This regional suitability model shows a higher suitability for WLBG in the region compared to Beauchamp et al.'s model (2013) but a more restricted result than the map created by the USDA (2012). While Beauchamp et al. found that only 1% of their study area was highly suitable (77,000 ha), our model predicted that 3.23% (258,036 ha**)** of this same area was highly suitable, which is over three times larger. The inclusion of 137 more presence points, possibly from a greater variety of site conditions, has likely allowed for a broader area to be deemed suitable, which is a cause for concern from a management perspective. However, a direct comparison cannot be made as our modeling approach was quite different from Beauchamp et al. despite using the same software (Maxent). Our results are also in contrast with those produced by the USDA's 2012 weed risk assessment suitability model, which uses plant hardiness, precipitation, and Köppen–Geiger climate classes from where the species occurs elsewhere in the world. Their model predicted that at the continental scale, approximately 30% of the United States is suitable habitat for WLBG (USDA, 2012). Therefore, while the predictions of our model suggest an expansion of WLBG's range in the eastern United States, the predicted area is much smaller than that predicted by the USDA. However, the USDA model did not include a multitude of other factors that may affect the presence of this species, such as topography. In addition, there is evidence that invasive plants can occupy niches in their introduced range that differ from those in their native ranges (Broennimann et al., 2004), highlighting the importance of utilizing presence points in the introduced range for ENMs for invasive species.

Several voices in the field of ecological niche modeling have increasingly called for incorporating model complexity and calibration that is often lacking in ENM studies (Cobos et al., 2019; Peterson et al., 2018; Warren & Seifert, 2011). In particular, identifying possible combinations of parameters for model selection can produce better fits to the data (Cobos et al., 2019; Spear, 1997) and can significantly affect performance (Warren & Seifert, 2011). We found support for these claims in this study, where statistical significance, AUC ratios, and omission rates differed widely between calibrated models (data not shown). In addition, our top ten models had regularization parameters ranging from 0.4 to 3.0 at the regional scale and 0.6 to 2.0 at the landscape scale. With regard to feature classes, all top ten models at the regional scale used lqpth or lqpt functions while the landscape models used lqp or lqpt functions. Therefore, while the Maxent graphical user interface automatically selects a regularization parameter (1.0) and feature classes (Phillips et al. 2006), these selections may not generate the best model.

Ecological niche models such as these can provide researchers and land managers with tools to study and respond to rapidly invading species such as WLBG. While ENM models such as Maxent have drawbacks, such as the difficulty in utilizing biological variables and transferring to new regions or times (Jarnevich & Reynolds, 2011; Warren & Seifert, 2011), these models have proven to be both insightful and high performing at landscape and regional scales (Elith et al., 2010; Jarnevich & Reynolds, 2011; Lemke et al., 2011; Liang et al., 2014). In addition, ENMs can reveal predictors associated with invasion that may not be visible at the local scale, such as the importance of climatic variables in this model. These results will be able to facilitate more effective monitoring and management at both the landscape and regional scales.

## CONFLICT OF INTEREST

None declared.

## AUTHOR CONTRIBUTION

**Anna K. M. Bowen:** Conceptualization (lead); Data curation (lead); Formal analysis (lead); Investigation (lead); Methodology (lead); Visualization (lead); Writing‐original draft (lead); Writing‐review & editing (lead). **Martin Henry Stevens:** Supervision (lead); Writing‐original draft (supporting); Writing‐review & editing (supporting).

## Supporting information

Table S1‐S3

## Data Availability

All scripts, raw data, and created environmental data are available on Dryad (http://doi:10.5061/dryad.kwh70rz10).
