# Supplementary material for: Temperature, topography, soil characteristics, and NDVI drive habitat preferences of a shade‐tolerant invasive grass
Source: Ecol Evol. 2020 Sep 23;10(19):10785–97. doi: 10.1002/ece3.6735 (PMC7548190; doi:10.1002/ece3.6735)
Supplement: Supplementary file 1 — Table S1‐S3 [file ECE3-10-10785-s001.docx]

**Table S1.** Predictor variables (N=38 for regional scale model, N=12 for landscape scale model) obtained. Variables include those dropped after determining collinearity (normal text), those dropped after preliminary model evaluation (italics), and variables that were used for final candidate model creation (bolded). Values in parentheses indicate the range and units for each predictor, with some predictors being unitless (e.g. NDVI).

| **Regional scale model** | **Landscape scale model** |
| --- | --- |
| **Annual mean temperature (6.1-15.8ºC)** |  |
| **Mean diurnal range (mean of monthly (max-min)) (6.8-14.3ºC)** |  |
| Isothermality (mean diurnal range / temperature annual range (*100)) (22.8-39.2ºC) |  |
| **Temperature seasonality (standard deviation *100) (717.9-980.8ºC)** |  |
| Max. temperature of warmest month (20.8-32.9ºC) |  |
| Min. temperature of the coldest month (-13.9-1.4ºC) |  |
| **Temperature annual range (28.3-39.4ºC)** |  |
| Mean temperature of wettest quarter (6.3-25.0ºC) |  |
| Mean temperature of driest quarter (-5.0-17.9ºC) |  |
| Mean temperature of warmest quarter (15.7-25.1ºC) |  |
| Mean temperature of coldest quarter (-5.5-6.6ºC) |  |
| **Annual precipitation (835-1462 mm)** |  |
| Precipitation of wettest month (90-156 mm) |  |
| Precipitation of driest month (14-106 mm) |  |
| **Precipitation seasonality (7.6-25.5ºC)** |  |
| Precipitation of wettest quarter (261-423 mm) |  |
| Precipitation of driest quarter (155-331 mm) |  |
| Precipitation of warmest quarter (255-398 mm) |  |
| Precipitation of coldest quarter (155-353 mm) |  |
| SOST (start of growing season time in day of the year) (-149-364) |  |
| *SOSN (start of season NDVI) (0.0-1.0)* |  |
| **EOST (end of growing season time in day of the year) (0-450)** |  |
| EOSN (end of season NDVI) (0.0-0.85) |  |
| *MAXT (day of year for maximum NDVI) (1-358)* |  |
| MAXN (maximum NDVI during season) (0.0-0.99) |  |
| DUR (length of growing season in number of days) (91-365) |  |
| AMP (difference between max NDVI and NDVI at start of season) (0.0-1.0) |  |
| **TIN (time-integrated NDNI, cumulative NDVI during growing season) (0.0-0.99)** |  |
|  | **NDVI (0.19-1.0)** |
| **Elevationation (0.0-1,731.0 m)** | **Elevationation (164.1-1,229.5 m)** |
| *Aspect (0-360 degrees)* | **Aspect (0-360 degrees)** |
| **Slope (0.0-33.1 degrees)** | **Slope (0.0-45.8 degrees)** |
| *Distance to streams (0.0-4,000.0 m)* | **Distance to streams (0.0-3,097.0 m)** |
| *Distance to roads (0.0-26,111.5 m)* | **Distance to roads (0.0-10,534.9 m)** |
|  | **Distance to trails (0.0-9,949.2 m)** |
| *Soil pH (4.0-7.9)* | **Soil pH (4.0-6.2)** |
| *Soil AWS (available water storage to 25 cm) (1.1-9.5 cm)* | **Soil AWS (available water storage to 25 cm) (1.23-5.2 cm)** |
| *Soil percent sand (0.0-99.4%)* | **Soil percent sand (20.5-83.5%)** |
| Soil percent silt (0.0-85.0%) | Soil percent silt (8.5-56.6%) |
| *Soil percent clay (0.0-70.0%)* | *Soil percent clay (7.5-50.0%)* |

Table S2. Top ten models for regional scale model, listed by increasing dAIC. Model information includes the predictors, regularization parameter (β), and feature class. The top model used for final model creation is bolded. Predictors in all other models that were included in the top model are bolded as well. It should be noted that the best model does not necessarily have the lowest dAIC because the evaluation process only accepts models with an omission rate of 0.05 or lower.

| Model predictors | β | Feature class | Mean AUC ratio | Omission rate | AICc | dAICc | # of parameters |
| --- | --- | --- | --- | --- | --- | --- | --- |
| **ann. mean temp., temp. seasonality, diurnal range, slope, TIN** | **0.6** | **lqpth** | **1.64** | **0.05** | **9917.93** | **173.78** | **95** |
| **ann. mean temp., temp. seasonality, diurnal range, slope, TIN** | 0.5 | lqpt | 1.66 | 0.05 | 9922.27 | 178.182 | 106 |
| **ann. mean temp., temp. seasonality, diurnal range, slope, TIN** | 0.6 | lqpt | 1.64 | 0.05 | 9922.41 | 178.25 | 96 |
| **ann. mean temp., temp. seasonality, diurnal range, slope, TIN** | 0.5 | lqpth | 1.65 | 0.05 | 9941.06 | 196.91 | 112 |
| **ann mean temp, diurnal range, TIN, slope**, ann precip, precip seasonality, EOST | 0.7 | lqpt | 1.72 | 0.05 | 9959.59 | 215.44 | 114 |
| **diurnal range, TIN, slope, temp season**, ann. precip., precip. seasonality, EOST, temp. ann. range | 0.6 | lqpt | 1.68 | 0.04 | 9964.19 | 220.04 | 124 |
| **diurnal range, TIN, slope, temp season**, ann. precip., precip. seasonality, EOST, temp. ann. range | 0.7 | lqpt | 1.66 | 0.04 | 9966.13 | 221.98 | 114 |
| **ann mean temp, diurnal range, TIN, slope**, EOST, precip seasonlity, temp ann range | 1.0 | lqpt | 1.69 | 0.05 | 9972.59 | 228.44 | 100 |
| **ann mean temp, tin, slope, temp seasonality**, precip seasonality | 0.8 | lqpt | 1.65 | 0.05 | 9983.11 | 238.96 | 95 |
| **ann mean temp, diurnal range, TIN, slope, temp seasonality**, elevationation, EOST, precip. seasonality | 3.0 | lqpth | 1.73 | 0.05 | 9987.90 | 243.75 | 66 |

Table S3. Top ten models for landscape scale model, listed by increasing dAIC. Model information includes the predictors, regularization parameter (β), and feature class. The top model used for final model creation is bolded. Predictors in all other models that were included in the top model are bolded as well.

| Model predictors | β | Feature class | Mean AUC ratio | Omission rate | AICc | dAICc | # of parameters |
| --- | --- | --- | --- | --- | --- | --- | --- |
| **AWS, dist. roads, dist. streams, dist. trails, elevation, NDVI, soil sand** | **0.9** | **lqpt** | **1.64** | **0.05** | **1886.54** | **0.00** | **28** |
| **AWS, dist. roads, dist. streams, dist. trails, elevation, NDVI, soil sand**, pH | 0.7 | lqp | 1.68 | 0.05 | 1890.03 | 3.48 | 18 |
| **AWS, dist. roads, dist. streams, dist. trails, elevation, NDVI, soil sand**, pH | 0.8 | lqp | 1.66 | 0.05 | 1890.23 | 3.69 | 17 |
| **AWS, dist. roads, dist. streams, dist. trails, elevation, NDVI, soil sand**, pH, slope | 2.0 | lqp | 1.68 | 0.05 | 1890.25 | 3.70 | 13 |
| **AWS, dist. roads, dist. streams, dist. trails, elevation, NDVI, soil sand**, pH | 0.6 | lqp | 1.67 | 0.05 | 1890.67 | 4.13 | 19 |
| **AWS, dist. roads, dist. streams, dist. trails, elevation, soil sand**, pH, slope | 0.9 | lqp | 1.63 | 0.05 | 1890.67 | 4.13 | 20 |
| **AWS, dist. roads, dist. streams, dist. trails, elevation, NDVI, soil sand** | 1.0 | lqpt | 1.64 | 0.05 | 1891.03 | 4.49 | 27 |
| **AWS, dist. roads, dist. streams, dist. trails, elevation, NDVI, soil sand** | 0.8 | lqpt | 1.63 | 0.05 | 1892.43 | 5.88 | 31 |
| **AWS, dist. roads, dist. streams, dist. trails, elevation, NDVI, soil sand**, pH, slope | 2.0 | lqpt | 1.70 | 0.05 | 1892.66 | 6.11 | 14 |
| **AWS, dist. roads, dist. streams, dist. trails, elevation, NDVI, soil sand**, aspect, pH, slope | 2.0 | lqp | 1.67 | 0.05 | 1892.70 | 6.15 | 14 |
